# Molecular prevalence and associated risk factors of *Entamoeba* spp. in donkeys in Shanxi Province, North China

**DOI:** 10.1186/s13071-025-06671-9

**Published:** 2025-02-05

**Authors:** Ze-Dong Zhang, Han-Dan Xiao, Dong-Yang Wang, Nan Su, Xun-Zhi Liu, Zi-Rui Wang, Shi-Chen Xie, Xing-Quan Zhu, Shuo Zhang, Wen-Wei Gao

**Affiliations:** 1 Laboratory of Parasitic Diseases, College of Veterinary Medicine, Shanxi Agricultural University, Taigu, Jinzhong, 030801 Shanxi People’s Republic of China; 2 The Yunnan Key Laboratory of Veterinary Etiological Biology, College of Veterinary Medicine, Yunnan Agricultural University, Kunming, 650201 Yunnan People’s Republic of China; 3 State Key Laboratory for Animal Disease Control and Prevention, Key Laboratory of Veterinary Parasitology of Gansu Province, Lanzhou Veterinary Research Institute, Chinese Academy of Agricultural Sciences, Lanzhou, 730046 Gansu People’s Republic of China

**Keywords:** *Entamoeba* spp., Donkeys, Prevalence, Zoonotic parasites, Shanxi Province

## Abstract

**Background:**

The intestinal protozoa *Entamoeba* spp. can infect humans and various animals, including donkeys, causing diarrhea and malabsorption and presenting significant risks to animal husbandry and public health. Most *Entamoeba* species are not pathogenic except for *Entamoeba histolytica*. China has among the highest rates of donkey farming worldwide. Donkey (*Equus asinus*) farming is increasingly important in China because of their draft and medicinal value; however, epidemiological data on *Entamoeba* spp. in donkeys remains limited globally. This study aimed to investigate the prevalence of *Entamoeba* in donkeys in Shanxi Province, North China, and assess associated risk factors using a molecular approach.

**Methods:**

Fecal samples of 815 donkeys from three representative geographical locations in Shanxi Province were collected to investigate the presence of *Entamoeba* spp. A portion of the small-subunit rRNA gene (SSU rRNA) was amplified and sequenced to determine the prevalence and species/genotypes of *Entamoeba* spp. Statistical analysis of possible risk factors was performed using Statistical Product and Service Solutions (SPSS) 26.0 software. The phylogenetic relationship of *Entamoeba* spp. was reconstructed using the neighbor-joining (NJ) method in Molecular Evolutionary Genetics Analysis (Mega) 7.0 software.

**Results:**

The overall prevalence of *Entamoeba* spp. in donkeys in Shanxi Province was 7.12% (58/815). Two species (*Entamoeba* sp. RL9 and *Entamoeba equi*) were identified by sequence analysis; of these, *Entamoeba* sp. RL9 was the most prevalent species in donkeys in this study. Statistical analysis revealed that the donkeys' sex, region, age, and altitude are the risk factors associated with *Entamoeba* spp. prevalence (*P* < 0.05). Phylogenetic analysis indicated that the sequences of *Entamoeba* sp. RL9 and *E. equi* isolated from donkeys in this study were clustered with previously reported animal-derived *Entamoeba* sp. RL9 and *E. equi* sequences, respectively.

**Conclusions:**

This study reports the occurrence and prevalence of *Entamoeba* spp. in donkeys worldwide for the first time to our knowledge. This not only expands the geographical distribution but also broadens the host range of *Entamoeba* spp., addressing the knowledge gap regarding the prevalence of *Entamoeba* spp. in donkeys, providing baseline data for carrying out prevention and control of *Entamoeba* spp. in donkeys in China.

**Graphical Abstract:**

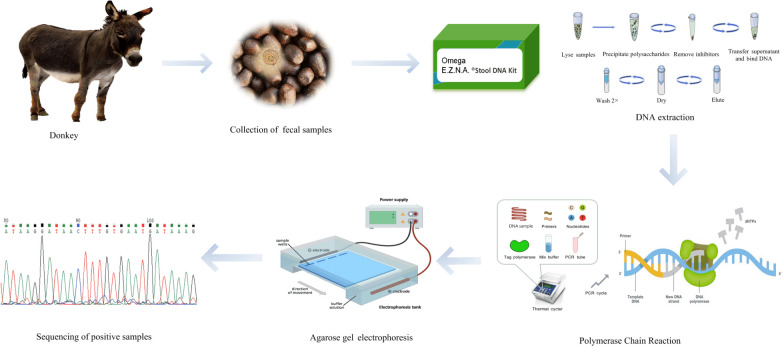

**Supplementary Information:**

The online version contains supplementary material available at 10.1186/s13071-025-06671-9.

## Background

*Entamoeba* spp. are zoonotic parasites which can infect humans and many animals, causing severe disease [1]. Ingestion of food or water contaminated with cysts and direct fecal-oral contact are the principal routes for *Entamoeba* infections in humans and animals, and the world′s overall prevalence of *Entamoeba* in humans was estimated to be 3.55% [1, 2]. Morphologically, *Entamoeba* species can be categorized into four groups: *Entamoeba gingivalis*-like group (species without cysts), *E. bovis*-like group (with uninucleated cysts), *E. histolytica*-like group (characterized by quadrinucleated cysts), and *E. coli*-like group (with octonucleated cysts) [3]. While most *Entamoeba* species are considered non-pathogenic, *E. histolytica* is known to cause significant intestinal disease, including hemorrhagic dysentery and liver abscesses, and can even lead to death [4, 5]. *Entamoeba histolytica* has also been detected in vegetables and fruits, which serve as the main sources of human nutritional intake, potentially threatening public health, especially among young children, the elderly, and immunocompromised individuals, who have weaker immune systems [6, 7]. The National Institute of Allergy and Infectious Diseases (NIAID) classified *E. histolytica* as a category B priority biodefense pathogen [8]. Furthermore, it also has been detected in river water in Iran, posing a significant threat to public health [9]. *Entamoeba* spp. has been detected in the feces of many animals, such as yaks and pigs [10, 11]. Notably, *E. histolytica* has been found in horse feces in China [12]. Previous studies estimated that *E. histolytica* affects around 50 million people worldwide, leading to approximately 100,000 deaths annually [13–15].

Generally, microscopy is a widely utilized diagnostic method in parasitology for identifying *Entamoeba* species [13]. However, *Entamoeba dispar* and *E. moshkovskii* cannot be morphologically differentiated from *E. histolytica* in either cyst or trophozoite form during microscopic examination [3, 4, 16]. Therefore, it is essential to implement molecular detection methods to accurately identify and differentiate *Entamoeba* species that exhibit overlapping morphological characteristics [3]. Currently, polymerase chain reaction (PCR) methods are extensively employed to detect and identify *Entamoeba* species, offering improved specificity and sensitivity [17, 18]. Due to the lack of morphological data for numerous new and known *Entamoeba* species, the concept of “ribosomal lineage” (RL) has been introduced to categorize sequences that diverge by > 5% from formerly described species, resulting in the identification of 11 RLs [10, 19].

The domestication of donkeys has substantially influenced human culture and the advancement of the livestock sector, playing a crucial role in economic and social realms throughout history [20]. Donkey meat is esteemed because of its high protein content, diverse unsaturated fatty acids, and essential trace elements [21]. Donkey milk as a specific nutrient supplement is rich in nutrients, including high levels of whey protein and vitamin C, which contribute to immune regulation and antioxidation [22, 23]. Donkey skin, abundant in collagen, is applied extensively in the food, pharmaceutical and cosmetic industries [24]. In China, donkey skin is the raw material of “Ejiao,” valued in the field of traditional medicine [25]. Nowadays, with the rapid development of agricultural modernization and rural urbanization, the use of donkeys is evolving from their traditional roles as draft animals and in meat and milk production to applications in medicine, cosmetics, and other functional bioproducts [26]. The global donkey husbandry industry has witnessed diverse trends and developments over the years. With increasing recognition of donkeys' multiple values, donkey farming is on the rise. Currently, the number of donkeys worldwide is 51.7 million, mainly distributed in Asia and Africa [27]. China ranks among the countries with the highest rates of donkey breeding worldwide, with a long history of almost 4000 years [28]. Shanxi Province, situated in north China, has a suitable habitat for donkey breeding due to unique natural conditions, vast mountains, abundant pasture resources and suitable climatic environment [29]. According to data, in 2019 Shanxi Province ranked seventh in China regarding the number of donkeys, with 113,700 donkeys [22].

Given the importance of donkey farming in Shanxi Province and China, investigation of *Entamoeba* spp. infection in donkeys is important to ensure the quality and quantity of donkey products. Nonetheless, there is currently a paucity of epidemiological data regarding *Entamoeba* spp. in donkeys worldwide. To date, there is only one report in which *E. histolytica* and *E. moshkovskii* were observed in horse feces from the Qinghai-Tibetan Plateau of China [12]. Therefore, the objectives of this study were to investigate the prevalence and genotypes of *Entamoeba* in donkeys in Shanxi Province and to assess related risk factors. The findings will not only expand the geographical distribution of *Entamoeba* but also broaden the understanding of host ranges, addressing the gap in global knowledge regarding the prevalence of *Entamoeba* spp. in donkeys and providing foundational information for the control and prevention of these parasites in the study regions.

## Methods

### Sampling collection

**Fig. 1:**
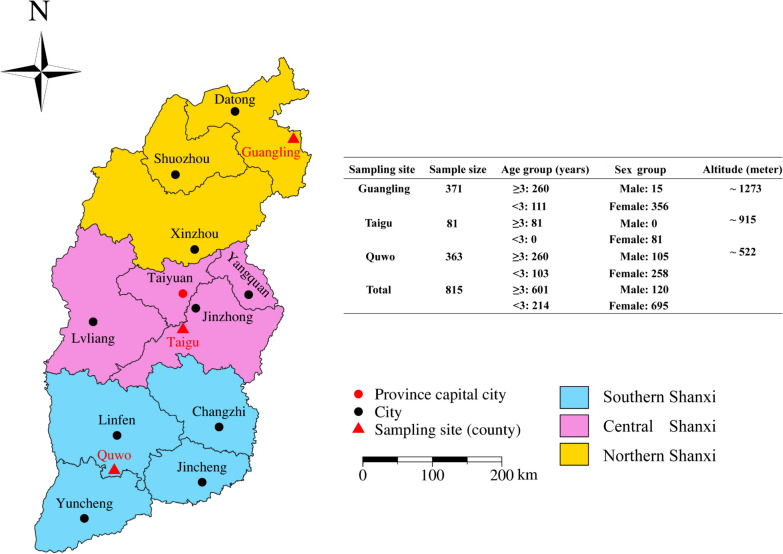
Sampling sites of donkey feces with sample numbers distribution and potential risk factors in Shanxi Province, North China

Between April and May 2023, a total of 815 fresh fecal samples were collected from donkeys across three representative cities in Shanxi Province, China: 81 from Jinzhong, 363 from Linfen, and 371 from Datong, representing the central, southern, and northern regions, respectively (Fig. 1). This study focused on randomly chosen donkeys of different ages at donkey farms in three representative regions. Any external contamination was avoided during the collection process. Freshly excreted, uncontaminated feces were collected immediately using disposable sterile gloves to ensure consistency in sample quality and labeled with pertinent details such as the donkey’s region, sex and age. No clinical symptoms such as diarrhea were found in any of the donkeys, all of which had no abnormal stool consistency. After collection, the samples were refrigerated with ice packs and transported to the Laboratory of Parasitic Diseases at Shanxi Agricultural University. All samples were stored at − 20 °C for further analysis. Altitude information was sourced from the NOAA’s National Center for Environmental Information (https://gis.ncdc.noaa.gov/maps/ncei/cdo/monthly, accessed on 15 July 2024).

### DNA extraction and PCR amplification

According to the manufacturer′s instructions, genomic DNA was extracted from 200 mg of each fecal sample using the commercial E.Z.N.A.^®^ Stool DNA Kit (Omega Bio-Tek Inc., Norcross, GA, USA) and stored at − 20 °C until used for molecular analysis. A portion (approximately 550 bp) of the small-subunit rRNA gene (SSU rRNA) was amplified by the primers Entam1 (5′-GTTGATCCTGCCAGTATTATATG-3′) and Entam2 (5′-CACTATTGGAGCTGGAATTAC-3′) [30]. A total of 25 µl PCR mixture was prepared for PCR amplification, including 2 µl dNTPs, 25 mM MgCl_2_, 2.5 µl 10 × PCR buffer (Mg^2+^ free), 0.25 µl of each primer, 1.25 U *Ex*-Taq (Takara, Dalian, China), 2 µl genomic DNA, and 16.25 µl ddH_2_O. PCR assay was performed according to a previous report [31]. To confirm amplification efficiency, each reaction included one positive control (verified DNA of *Entamoeba* sp. by sequencing) and one negative control (sterile, enzyme-free water). The PCR products were subjected to electrophoresis on 1.5% agarose gels for 35 min at a constant voltage of 120 V stained with ethidium bromide and visualized under UV transillumination.

### Sequencing and phylogenetic analysis

All successfully amplified PCR products with expected fragment size were sent to Sangon Biotech Co. Ltd. (Shanghai, China) for bidirectional sequencing. The resulting sequences were proofread and assembled using Chromas V1.3 software. To determine the species/genotypes of *Entamoeba* spp., the Basic Local Alignment Search Tool (BLAST) was applied to align the DNA sequences with previously reported DNA sequences of *Entamoeba* spp. The phylogenetic relationships among *Entamoeba* spp. were reconstructed using the neighbor-joining (NJ) method and Kimura 2-parameter model in MEGA 7.0 software. To assess branch reliability, bootstrap analysis was performed with 1000 replicates.

### Statistical analysis

The chi-square (*χ*^2^) test in SPSS 26.0 (SPSS Inc., Chicago, IL, USA) was used to evaluate the correlation between the prevalence and risk factors (regions, ages, sexes, and altitudes) of *Entamoeba* spp. In addition, the correlation between risk factors and the identified species was analyzed. Odds ratios (ORs) and 95% confidence intervals (95% CIs) were calculated to determine the strength of correlation between prevalence and tested variables. The difference was considered to be significant when the *P*-value was < 0.05.

## Results

### Prevalence and species of *Entamoeba* spp. in donkeys

**Fig. 2:**
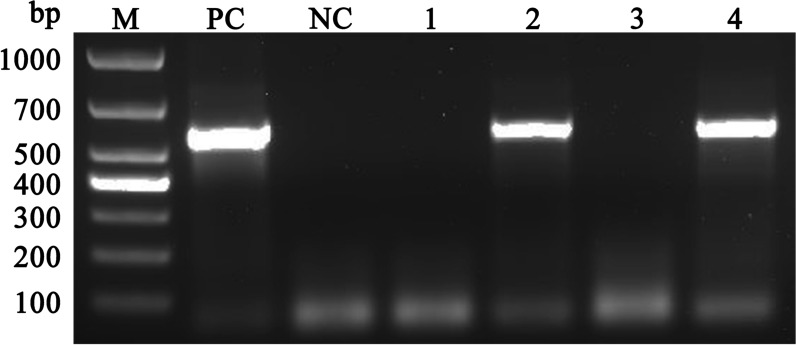
Agarose gel electrophoresis of the PCR products. Lane M: 1000-bp DNA ladder; lane PC: positive control; lane NC: negative control; lanes 1–4: PCR amplification with primers specific for a 550-bp fragment of the *Entamoeba* spp. SSU rRNA gene using the genomic DNA extracted from each donkey fecal sample as a template

As illustrated in Fig. 2, PCR amplification yielded specific bands of the expected sizes (approximately 550 bp). Based on SSU rRNA gene, 58 of 815 fecal samples (7.12%, 95% CI 5.35–8.88%) tested positive for *Entamoeba* by PCR amplification in this study. As shown in Table 1, the highest prevalence of *Entamoeba* in donkeys was identified in Jinzhong City (49.38%, 95% CI 38.49–60.27%), which was significantly higher than in the other two sampling districts (*P* < 0.001). Statistical analysis revealed that significant differences in the prevalence of *Entamoeba* were found among sex groups (*P* < 0.001), age groups (*P* = 0.004), region groups (*P* < 0.001) and altitude groups (*P* = 0.004).

**Table 1:** Risk factors related to the prevalence of *Entamoeba* spp. in donkeys in Shanxi Province, North China

Risk factor	Category	Positive/tested (no.)	Prevalence % (95% CI)	OR (95% CI)	*P*-value	Species (no.)
Age	< 3 years	6/214	2.8 (0.59–5.02)	Ref		*Entamoeba* sp. RL9 (6)
	≥ 3 years	**52/601**	**8.65 (6.40–10.91)**	**3.28 (1.39–7.76)**	**0.004**	*Entamoeba* sp. RL9 (49), *E. equi* (3)
Region	Jinzhong	**40/81**	**49.38 (38.49–60.27)**	**176.10 (41.04–755.54)**		*Entamoeba* sp. RL9 (40)
	Linfen	2/363	0.55 (0.00–1.31)	Ref		*Entamoeba* sp. RL9 (2)
	Datong	**16/371**	**4.31 (2.25–6.38)**	**8.14 (1.86–35.64)**	** < 0.001**	*Entamoeba* sp. RL9 (13), *E. equi* (3)
Sex	Male	0/120	0	–		–
	Female	**58/695**	**8.35 (6.29–10.40)**	–	** < 0.001**	*Entamoeba* sp. RL9 (55), *E. equi* (3)
Altitude	> 1000 m	16/371	4.31 (2.25–6.38)	Ref		*Entamoeba* sp. RL9 (13), *E. equi* (3)
	≤ 1000 m	**42/444**	**9.46 (6.74–12.18)**	**2.32 (1.28–4.20)**	**0.004**	*Entamoeba* sp. RL9 (42)
Total		58/815	7.12 (5.35–8.88)			*Entamoeba* sp. RL9 (55), *E. equi* (3)

Values showed in bold represent statistical significance with *P* < 0.05

Notably, no *Entamoeba*-positive samples were identified in male donkeys. In this study, *Entamoeba* sp. RL9 and *Entamoeba equi* were identified among 58 positive samples, with *Entamoeba* sp. RL9 being the predominant species (94.83%, 55/58). Additionally, *E. equi* (5.17%, 3/58) was found exclusively in female donkeys aged ≥ 3 years in Datong City.

### Species and genotype distribution of *Entamoeba* spp. in donkeys

Significant difference in the prevalence of *Entamoeba* sp. RL9 in donkeys was found among sex groups (*P* = 0.033), age groups (*P* = 0.011), regional groups (*P* < 0.001), and altitude groups (*P* < 0.001) (Additional file 1: Table S1). In addition, only three positive samples were identified as *E. equi*, and no significant difference was found between the prevalence of *Entamoeba equi* and related risk factors.

### Population genetic analyses of *Entamoeba* spp.

**Fig. 3:**
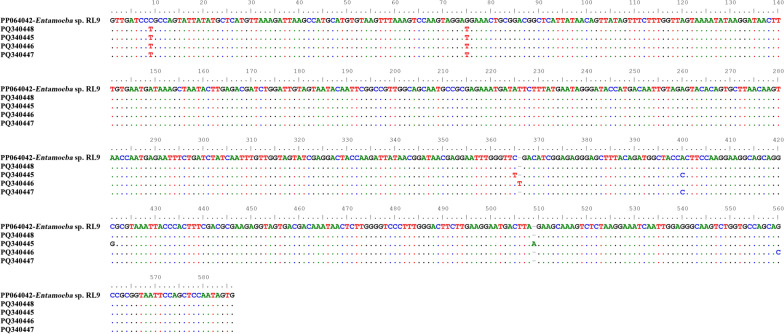
Alignment of the SSU rRNA sequences of *Entamoeba* sp. RL9 isolates acquired in the current study (PQ340445–PQ340448) and that of an *Entamoeba* sp. RL9 isolate from a previous study (PP064062). Dashes represent base deficiency; dots represent base identical to the reference sequences

In this study, 58 *Entamoeba*-positive samples were successfully sequenced based on SSU rRNA gene, and seven distinct representative *Entamoeba* sequences in donkeys (including 4 sequences of *Entamoeba* sp. RL9 and 3 sequences of *E. equi*) were acquired. The four *Entamoeba* sp. RL9 sequences shared 98.97–99.66% identity with the reference strain (accession no. PP064062) (Fig. 3). Similarly, the three *E. equi* sequences showed 97.09–99.66% similarity to the reference sequence isolated from a horse in the UK (accession no. DQ286371). Sequence analysis of these seven sequences indicated that the *Entamoeba* sp. RL9 sequences exhibited slight allelic variations compared with the *E. equi* sequences isolated from donkeys in this study. The representative sequences acquired in this study were deposited in GenBank database with the following accession numbers: PQ340445 to PQ340448 for *Entamoeba* sp. RL9 and PQ340441 to PQ340443 for *E. equi*.

Moreover, the average genetic divergence within the *Entamoeba* sp. RL9 sequences was found to be fivefold smaller than the divergence observed within the *E. equi* sequences. The nucleotide difference in the partial SSU rRNA sequences between the two identified *Entamoeba* species was significant, with mean differences of 92 nucleotides (15.73%) (Additional file 2: Table S2).

**Fig. 4:**
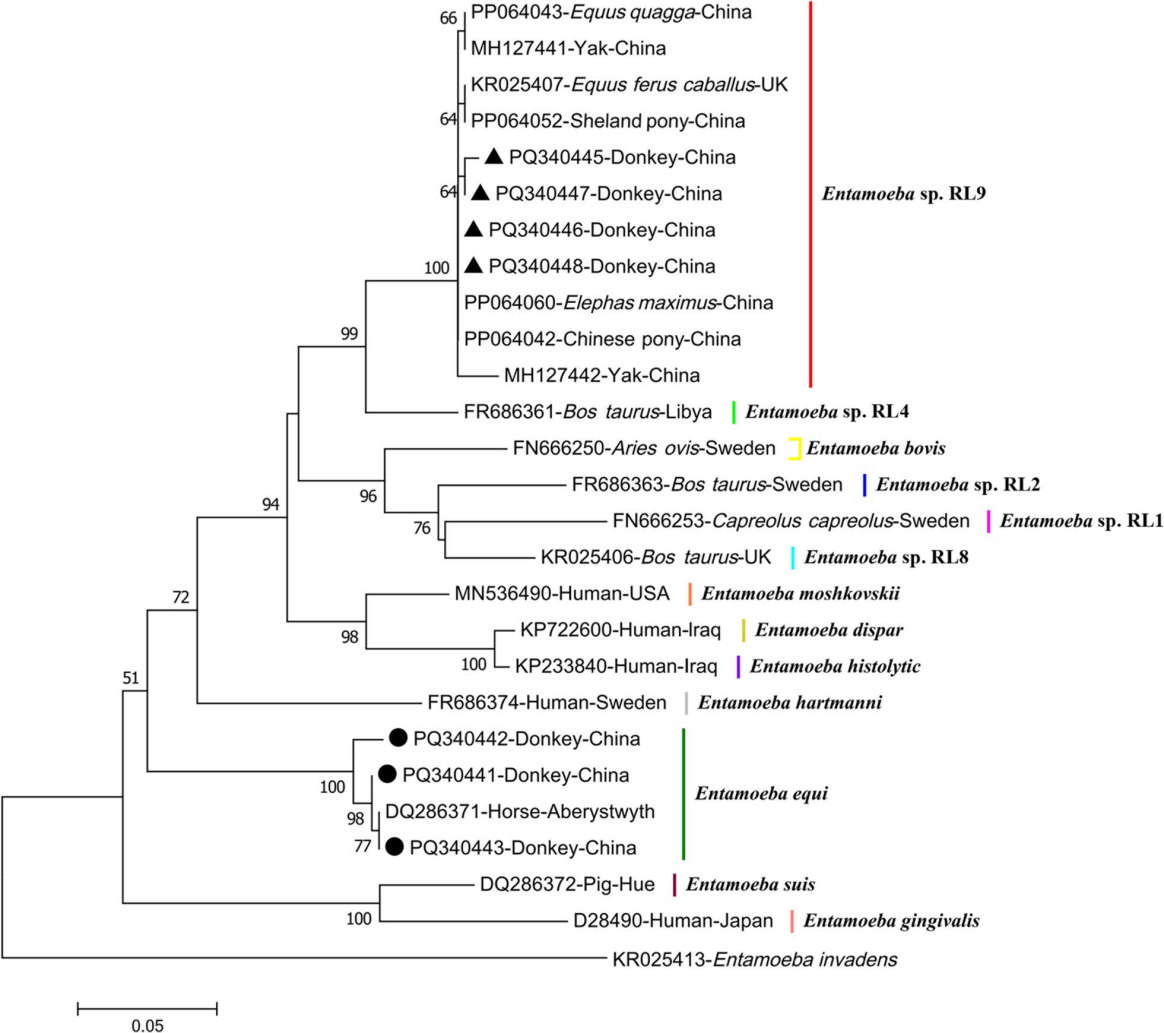
Phylogenetic tree of the obtained sequences (*Entamoeba* sp. RL9 marked with a triangle and *Entamoeba equi* marked with a black circle) in the present study and previously reported sequences of *Entamoeba* spp. based on SSU rRNA. The Kimura2-parameter model method was used with bootstrap evaluation of 1000 replicates. Bootstrap values are shown when > 50%

As shown in Fig. 4, phylogenetic analysis indicated that the sequences of *Entamoeba* sp. RL9 and *E. equi* isolated from donkeys in this study were gathered in a branch with previously reported animal-derived *Entamoeba* sp. RL9 and *E. equi* sequences, respectively. Furthermore, the tree showed that *Entamoeba* sp. RL9 had a close relationship with *Entamoeba* sp. RL4, consistent with a previous report [13].

## Discussion

With the development of modern agricultural and transportation machines, the transportation value of donkeys has gradually declined; however, their commercial value has been increasing. Donkey products have rich nutritional and medicinal value and are favored by many people. Donkey farming is becoming increasingly important in China [32].

Gastrointestinal parasites (GIPs) threaten animal health and impact the development of animal husbandry [33]. *Entamoeba* spp. are common intestinal protozoa that infect humans and animals, leading to symptoms such as diarrhea, weight loss, and decreased feeding efficiency, which can result in considerable economic losses [34–36]. *Entamoeba histolytica*, the causative agent of amebiasis, is a potentially pathogenic threat to humans and animals, causing dysentery and liver abscesses [37]. However, to the best of our knowledge, worldwide, no data on the prevalence of *Entamoeba* spp. in donkeys were available prior to the present study. This study aimed to characterize the prevalence and species/genotype of *Entamoeba* in donkeys in Shanxi Province, north China, utilizing a molecular method to assess zoonotic transmission potential and the further investigate the genetic diversity of *Entamoeba* in donkeys. To our knowledge, this is the first report identifying *Entamoeba* species in donkeys using a molecular method. Importantly, *E. histolytica*, the pathogen responsible for human amoebiasis, was not detected in this study.

Based on the SSU rRNA gene of *Entamoeba* spp., the overall prevalence of *Entamoeba* in donkeys in Shanxi province was 7.12% (58/815). In contrast to data on alpacas in Shanxi Province, the low *Entamoeba* spp. prevalence in the examined donkeys could be attributed to different species, sampling times, and locations [31]. In addition, compared to yaks and other farm animals [10, 12], donkeys on donkey farms had better living conditions, which may have reduced their risk of *Entamoeba* spp. infection.

Using microscopy, Kareem et al. examined *Entamoeba* in donkeys in southeastern Iran but found no positive samples [34]. Among *Equids*, which include horses, donkeys, mules, and hinnies, donkeys and horses are closely related and share similar parasite species [38]. In contrast to no reports of *Entamoeba* infection in donkeys in China, horses on the Qinghai-Tibetan Plateau were reported to be infected with *Entamoeba*, where genotype *Entamoeba* sp. RL9 was also identified [12]. These findings suggest that donkeys might act as potential reservoirs for *Entamoeba*, broadening our understanding of *Entamoeba* multiplicity and expanding the host range. Statistical analysis showed that significant differences in the *Entamoeba* prevalence in donkeys were detected among age, sex, region, and altitude groups. Considering three regions, the highest *Entamoeba* prevalence in donkeys was detected in Jinzhong City (49.38%, 40/81), followed by Datong City (4.31%, 16/371) and Linfen City (0.55%, 2/363). The elevated prevalence in Jinzhong City may be influenced by its geographic proximity to Taiyuan, Shanxi Province′s largest transportation hub and population center. Additionally, the prevalence of *Entamoeba* spp. in donkeys had a significant correlation with donkey age (*P* < 0.05) in this study, showing a higher prevalence in donkeys aged ≥ 3 years; however, this is inconsistent with a previous study which detected *Entamoeba* in monkeys [39]. This discrepancy may stem from the greater attention often given to younger animals because of their comparatively weaker immune systems, whereas older animals were assumed to have stronger immunity and might have received less attention [11]. Altitude also appeared to impact *Entamoeba* transmission, with 72.4% (42/58) of positive samples being found in areas < 1000 m. Notably, *Entamoeba*-positive samples were found exclusively in female donkeys, which is different from results of an earlier study in humans in Egypt where males had significantly higher *Entamoeba* prevalence than females [40]. In addition, we also analyzed the correlation between risk factors and the identified *Entamoeba* species. Statistical analysis revealed that significant differences in *Entamoeba* sp. RL9 prevalence were found in sex, age, region, and altitude groups (*P* < 0.05). However, no significant difference was found between *E. equi* prevalence and the related risk factors. These findings highlight the need for further epidemiological studies involving larger sample sizes and broader geographic locations to better understand the distribution and transmission of *Entamoeba* spp.

Phylogenetic analysis demonstrated that two species/genotypes identified in this study, namely *Entamoeba* sp. RL9 and *E. equi*, were clustered into their corresponding branches of species/genotypes. *Entamoeba* sp. RL9 was the predominant genotype (94.8%, 55/58), with significantly higher than previously reported rates in horses (20%, 3/15) and donkeys (0%, 0/2) from Devon, UK, as well as in yaks (0.54%, 2/373) and horses (6.25%, 2/32) from Qinghai Province, China [10, 12, 41]. Notably, *Entamoeba* sp. RL9 has been identified in various other animal species, whereas *E. equi* has only been detected in equines such as horses and zebras [19, 42].

Infection of humans and animals with *Entamoeba* spp. has become increasingly important globally; however, there are still significant challenges to the treatment of *Entamoeba* infection. Currently, nitroimidazole is the primary treatment for amoebiasis; thus, alternative therapies are necessary because of concerns about toxicity and potential drug resistance [2].

*Entamoeba histolytica* and *E. moshkovskii* can cause severe diseases, which have been identified in horses, indicating that donkeys may also act as potential hosts for zoonotic transmission. However, due to the limitation of sampling quantities and sampling locations, further studies are warranted to better understand the factors influencing *Entamoeba* prevalence in donkeys by expanding both the geographical locations and sampling sizes, which in turn will lead to a deeper understanding of the pathogenicity of *Entamoeba* and the role of donkeys in its transmission.

## Conclusions

This study revealed the prevalence and genotypes of *Entamoeba* spp. in donkeys in Shanxi Province for the first time to our knowledge. Two genotypes/species (*Entamoeba* sp. RL9 and *E. equi*) were detected in this study. These findings not only address the gap in global knowledge regarding the prevalence of *Entamoeba* spp. in donkeys but also broaden the understanding of host ranges and geographical distribution, offering essential baseline data for the prevention and control of *Entamoeba* spp. in donkeys in Shanxi Province. Future studies are necessary to examine *Entamoeba* spp. and clarify its potential effect on the health of donkeys and humans. Implementation of better management measures on donkey farms to decrease *Entamoeba* spp. infections in donkeys is suggested.

## Supplementary Information

Additional file 1: Table S1. Risk factors related to *Entamoeba* sp. RL9 in donkeys in Shanxi Province, North China.

Additional file 2: Table S2. Mean sequence divergence and number of differences (nucleotides) between* Entamoeba* sp. RL9 and *Entamoeba equi *sequences within clades.

## Data Availability

No datasets were generated or analyzed during the current study.

## Abbreviations

SSU rRNA: Small subunit ribosomal RNA
SPSS: Statistical Product and Service Solutions
Mega: Molecular Evolutionary Genetics Analysis
NIAID: National Institute of Allergy and Infectious Diseases
PCR: Polymerase chain reaction
BLAST: Basic Local Alignment Search Tool
OR: Odds ratio
CI: Confidence interval
RL: Ribosomal lineage
NJ: Neighbor joining
