# Supplementary material for: Molecular prevalence and associated risk factors of Entamoeba spp. in donkeys in Shanxi Province, North China
Source: Parasit Vectors. 2025 Feb 5;18:42. doi: 10.1186/s13071-025-06671-9 (PMC11796150; doi:10.1186/s13071-025-06671-9)
Supplement: Supplementary file 1 — Additional file 1: Table S1. Risk factors related to Entamoeba sp. RL9 in donkeys in Shanxi Province, North China. [file 13071_2025_6671_MOESM1_ESM.docx]

**Additional file 1: Table S1.** Risk factors related to *Entamoeba* sp.RL9 in donkeys in Shanxi Province, North China.

| Species | Risk Factor | Category | No. of Positive | No. of Tested | *P*-Value |
| --- | --- | --- | --- | --- | --- |
| *Entamoeba* sp.RL9 | Age | < 3 years | 6 | 214 |  |
|  |  | ≥ 3 years | **49** | **601** | **0.011** |
|  | Region | Jinzhong | **40** | **81** | **< 0.001** |
|  |  | Linfen | 2 | 363 |  |
|  |  | Datong | **13** | **371** |  |
|  | Sex | Male | 0 | 120 |  |
|  |  | Female | **55** | **695** | **0.033** |
|  | Altitude | > 1000 m | 13 | 371 |  |
|  |  | ≤ 1000 m | **42** | **444** | **< 0.001** |
|  | Total |  | 55 | 815 |  |

Values showed in bold represent the statistical significance with *P* < 0.05 level.
