# Supplementary material for: Molecular prevalence and associated risk factors of Entamoeba spp. in donkeys in Shanxi Province, North China
Source: Parasit Vectors. 2025 Feb 5;18:42. doi: 10.1186/s13071-025-06671-9 (PMC11796150; doi:10.1186/s13071-025-06671-9)
Supplement: Supplementary file 2 — Additional file 2: Table S2. Mean sequence divergence and number of differences (nucleotides) between Entamoeba sp. RL9 and Entamoeba equi sequences within clades. [file 13071_2025_6671_MOESM2_ESM.docx]

**Additional file 2: Table S2.** Mean sequence divergence and number of differences (nucleotides) between *Entamoeba* sp. RL9 and *Entamoeba* *equi* sequences within clades.

| Clades | Sequence divergence % | Number of differences |
| --- | --- | --- |
| *Entamoeba* sp. RL9 VS *Entamoeba* *equi* | 15.85 ± 0.23 | 92.75 ± 1.36 |
| Within *Entamoeba* sp. RL9 | 0.51 ± 0.24 | 3.00 ± 1.41 |
| Within *Entamoeba* *equi* | 2.45 ± 0.10 | 14.33 ± 4.62 |
